# A Convexity-Preserving Level-Set Method for the Segmentation of Tumor Organoids

**DOI:** 10.3390/bioengineering11060601

**Published:** 2024-06-12

**Authors:** Xiaoyi Lei, Luying Gui, Hairong Liu

**Affiliations:** 1 School of Science, Nanjing Forestry University, Nanjing 210037, China; 2 School of Mathematics and Statistics, Nanjing University of Science and Technology, Nanjing 210094, China

**Keywords:** convexity, image segmentation, tumor organoid, level set

## Abstract

Tumor organoid cultures play a crucial role in clinical practice, particularly in guiding medication by accurately determining the morphology and size of the organoids. However, segmenting individual tumor organoids is challenging due to their inhomogeneous internal intensity and overlapping structures. This paper proposes a convexity-preserving level-set segmentation 4 model based on the characteristics of tumor organoid images to segment individual tumor organoids precisely. Considering the predominant spherical shape exhibited by organoid growth, we propose a level-set model that includes a data-driven term, a curvature term, and a regularization term. The data-driven term pulls the contour to the vicinity of the boundary; the curvature term ensures the maintenance of convexity in the targeted segmentation, and the regularization term controls the smoothness and propagation of the contour. The proposed model aids in overcoming interference from factors such as overlap and noise, enabling the evolving curve to converge to the actual boundary of the target accurately. Furthermore, we propose a selectable and targeted initialization method that guarantees precise segmentation of specific regions of interest. Experiments on 51 pancreatic ductal adenocarcinoma organoid images show that our model achieved excellent segmentation results. The average Dice value and computation time are 98.81±0.48% and 20.67 s. Compared with the C-V and CPLSE models, it is more accurate and takes less time.

## 1. Introduction

In the past two decades, organoid technology has emerged as a promising advancement in clinical treatment. Organoid is a collection of organ-specific cell types that develops from stem cells or organ progenitors and self-organizes through cell sorting and spatially restricted lineage commitment like in vivo [1]. In 2011, Sato et al. took the lead in establishing colon adenomatous organoids [2]. Establishing the tumor organoid model is another significant breakthrough in the organoid field, providing a reliable cancer research and treatment model. So far, many tumor organoids have been successfully cultured, including prostate cancer [3], pancreatic cancer [4], ovarian cancer [5], etc.

Tumor organoids can simulate the structure and function of tumors in vivo to the greatest extent, which can directly display the process of tumor growth [6]. It is essential to observe the shape and size of tumor organoids, which can be used to screen anti-tumor drugs or study the mechanism of tumor genesis and development. Therefore, it is of practical significance to realize the segmentation of tumor organoid images, which can help us observe the changes in tumor organoids more clearly.

Up to now, more research is needed on the segmentation of organoid images. By cooperating with professional doctors, we have some images of pancreatic ductal adenocarcinoma organoids in our hands. During the segmentation experiment, we found the following difficulties: (1) intensity inhomogeneity inside of the organoid, which makes it difficult to segment the organoid boundary when the initial contour line is located in an internal area; (2) overlapping organoids: overlapping parts will have non-negligible impacts on the segmentation results; (3) image noise; (4) the limitations of imaging devices. Organoids that are not on the focal plane can be very blurry.

Existing image-segmentation methods include traditional segmentation methods [7,8] and deep learning segmentation methods [9,10]. Although deep learning segmentation methods are more efficient and intelligent, they require a large amount of data for training to improve the generalization ability. Small sample images are more suitable for traditional segmentation methods. Active contour models (ACMs) are a typical traditional segmentation method, widely used in image segmentation in the past few decades. The existing ACMs can be divided into two major classes: edge-based models [11,12,13] and region-based models [14,15,16]. Edge-based models apply local edge and gradient information to evolve the initial contour to the object boundaries. Region-based models employ regional statistical information to guide the motion of the contour. However, the ACMs may fail to segment some complex images, for instance due to intensity inhomogeneity. Furthermore, higher level information can be incorporated into the ACMs, such as shape priors [17,18], Euler elasticity energy [19,20], and convexity preservation [21,22].

In recent years, Wang et al. developed a res-double dynamic conv attention U-Net model targeting bladder cancer organoids [23]. Lefferts et al. proposed a MASK-RCNN allowing for the segmentation of individual intestinal patient-derived organoid structures from bright field images [24]. They both focus on the overall segmentation of organoid images and lack precise segmentation of individual organoids. In this paper, we mainly focus on segmenting single tumor organoids. With the segmentation result, we can further observe the morphology and size of individual tumor organoids to guide clinical treatment. Through observing the organoid images presented in academic papers [25,26] and our possession, we found that the intensity inside the organoids is extremely inhomogeneous, while the background is relatively homogeneous. We plan to locate the initial contour outside of the single organoid to be segmented and shrink it inward to the object boundary. At the same time, it is necessary to ensure the contour’s convexity-preserving ability for the complete segmentation of tumor organoid boundary, especially in the overlapping areas of organoids. Combined with the convexity-preserving mechanism of the curves, we propose a level-set model for tumor organoid image segmentation. It mainly solves the difficulty of the single segmentation of overlapping tumor organoids. On the other hand, we propose an automatic initialization method, which speeds up and guarantees the implementation of the segmentation process.

The rest of this paper is organized as follows. Section 2 will review the C-V and CPLSE models associated with the proposed model. In Section 3, we present the proposed model. In Section 4, we show the principle and process of automatic initialization in detail. In Section 5, we use 51 images to show the segmentation results of our model and compare the accuracy with the other two methods. Finally, the discussion and conclusion are given in Section 6.

## 2. The Related Works

### 2.1. The C-V Model

Chan and Vese [14] proposed an ACM based on the simplified Mumford and Shah model [27] and the level-set method. Let Ω be a bounded open subset of $R^{2}$, and *I*: Ω→R is the input image. Under the level-set framework, the closed contour C⊂Ω is represented by the zero level-set function ϕ(x), such that

(1) $$\left\{\begin{array}{c} \{C=x\in \Omega :\phi (x)=0\},\\ \{inside(C)=x\in \Omega :\phi (x)>0\},\\ \{outside(C)=x\in \Omega :\phi (x)<0\}, \end{array}\right.$$

Then, the energy functional of the C-V model can be formulated as

(2) $$\begin{array}{cc} E^{CV}= & \mu \int_{\Omega }\delta_{\epsilon }\left(\phi \left(x\right)\right)\left|\nabla \phi \right|dx+v\int_{\Omega }H_{\epsilon }\left(\phi \left(x\right)\right)dx+\lambda_{1}\int_{\Omega }{\left(I-c_{1}\right)}^{2}H_{\epsilon }\left(\phi \left(x\right)\right)dx\\ \; & +\lambda_{2}\int_{\Omega }{\left(I-c_{2}\right)}^{2}\left(1-H_{\epsilon }\left(\phi \left(x\right)\right)\right)dx \end{array}$$

where μ≥0, v≥0, $\lambda_{1}>0$, and $\lambda_{2}>0$ are fixed parameters and $c_{1}$ and $c_{2}$ are the average intensities inside and outside the contour ϕ. $H_{\epsilon }\left(\phi \left(x\right)\right)$ is the Heaviside function, and the Dirac delta function $\delta_{\epsilon }\left(\phi \left(x\right)\right)$ is the derivative of $H_{\epsilon }\left(z\right)$. Keep $c_{1}$ and $c_{2}$ fixed, and minimize $E^{CV}$ with respect to using the standard gradient descent method by solving the gradient flow equation as follows

(3) $$\frac{\partial \phi }{\partial t}=\delta_{\epsilon }\left(\phi \right)\mu \mathrm{div}\left(\frac{\nabla \phi }{\left|\nabla \phi \right|}\right)-v\delta_{\epsilon }\left(\phi \right)-\lambda_{1}\delta_{\epsilon }\left(\phi \right){\left(I-c_{1}\right)}^{2}+\lambda_{2}\delta_{\epsilon }\left(\phi \right){\left(I-c_{2}\right)}^{2}$$

The C-V model is a typical region-based model that utilizes global intensity information inside and outside the contour ϕ. However, for images with segmentation problems such as intensity inhomogeneity, it is difficult to achieve satisfactory results from the C-V model.

### 2.2. The CPLSE Model

Li and Shi [28] proposed a level-set method with a convexity-preserving mechanism, for the segmentation of the cardiac left ventricle, which is desired to be convex and include the cavity, trabeculae, and papillary muscles. It can be deduced that ϕ is convex at the pixels where κ≥0 and concave where κ<0 [29]. Therefore, the curvature sign indicator function (CSI) can be defined by

(4) $$s\left(\kappa \right)=\left\{\begin{array}{cc} 1, & \kappa \geq 0\\ 0, & \kappa <0 \end{array}\right.$$

Combined with the s(κ) function, the specific level-set evolution is as follows

(5) $$\frac{\partial \phi }{\partial t}=s\left(\kappa \right)D\left(\phi ;I\right)+\left(1-s\left(\kappa \right)\right)\kappa \delta_{\epsilon }\left(\phi \right)+R\left(\phi \right)$$

where D(ϕ;I) and R(ϕ) are defined based on the distance-regularized level-set evolution (DRLSE) model in [30] by

(6) $$D\left(\phi ;I\right)=\lambda \delta_{\epsilon }\left(\phi \right)div\left(g\frac{\nabla \phi }{\left|\nabla \phi \right|}\right)+\alpha g\delta_{\epsilon }\left(\phi \right)$$

and

(7) $$R\left(\phi \right)=\mu div(d_{p}\left(|\nabla \phi |)\nabla \phi \right)$$

where p(s) is the double-well potential defined by

(8) $$p\left(s\right)=\left\{\begin{array}{cc} \frac{1}{{\left(2\pi \right)}^{2}}\left(1-cos\left(2\pi s\right)\right), & s\leq 1\\ \frac{1}{2}{\left(s-1\right)}^{2}, & s>1 \end{array}\right.$$

and

(9) $$d_{p}\left(s\right)≜\frac{p^{\prime }\left(s\right)}{s};$$

the corresponding convexity-preserving level-set evolution (CPLSE) model can be expressed as follows

(10) $$\frac{\partial \phi }{\partial t}=\mu div(d_{p}\left(|\nabla \phi |)\nabla \phi \right)+\lambda s\left(\kappa \right)\delta_{\epsilon }\left(\phi \right)div\left(g\frac{\nabla \phi }{\left|\nabla \phi \right|}\right)+\alpha s\left(\kappa \right)g\delta_{\epsilon }\left(\phi \right)+\left(1-s\left(\kappa \right)\right)\kappa \delta \left(\phi \right)$$

The CPLSE model incorporates the sign of the curvature of ϕ and the DRLSE model to control the curve evolution, in order to obtain the convex segmentation result of the cardiac left ventricle.

## 3. The Proposed Model

**Figure 1:**
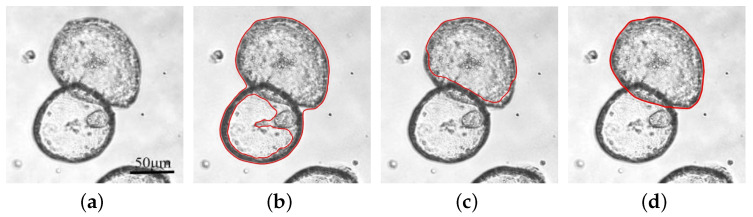
Organoid convexity-preserving segmentation. (**a**) Original image. (**b**) Segmentation result using CV model with consistent internal and external weights. (**c**) Segmentation result using CV model with high internal weight. (**d**) The desired result with convexity-preserving model. The red lines show the position of the contours.

Organoids are self-organized cellular clusters that tend to be smoothly round or oval. Figure 1a is an image of pancreatic ductal adenocarcinoma organoids. There are two partially overlapping tumor organoids. The lower organoid is nearly round, while the upper one is nearly oval. Take the segmentation of the upper organoid as an example. Directly based on the C-V model with the same internal and external weights, the segmentation results will be largely affected by the lower organoid’s internal inhomogeneous areas, as shown in Figure 1b. On the other hand, when the internal weight of the C-V model is increased, the contour will shrink towards the boundary of the upper organoid, but it will break through the boundary, while the upper one cannot be completely separated, as shown in Figure 1c. The desired result should be a continuous and convex shape, as shown in Figure 1d. Therefore, it is necessary to introduce a convexity-preserving method of the segmentation model to ensure convex and satisfactory segmentation results. Given that, we propose a novel level-set evaluation algorithm as follows:

(11) $$\frac{\partial \phi }{\partial t}=s\left(\kappa \right)\cdot F\left(\phi ,c_{1},c_{2}\right)+\alpha \cdot L\left(\phi \right)+\mu \cdot A\left(\phi \right)+\beta \cdot \left(1-s\left(\kappa \right)\right)\cdot V\left(\phi \right)$$

where $F(\phi ,c_{1},c_{2})$ is the Data-driven term, and it is defined by

(12) $$F\left(\phi ,c_{1},c_{2}\right)=\left[\lambda_{1}{\left(I-c_{1}\right)}^{2}-\lambda_{2}{\left(I-c_{2}\right)}^{2}\right]\delta_{\epsilon }\left(\phi \right)$$

where $\lambda_{1}$ > 0 and $\lambda_{2}$ > 0 are constants that represent the internal and external weights. $c_{1}$ and $c_{2}$ are the average intensities inside and outside the contour ϕ. $H_{\epsilon }\left(\phi \left(x\right)\right)$ is the Heaviside function, and the Dirac delta function $\delta_{\epsilon }\left(\phi \left(x\right)\right)$ is the derivative of $H_{\epsilon }\left(z\right)$. α>0, μ>0, and β>0 are the coefficients of the length term L(ϕ), area term A(ϕ), and curvature term V(ϕ). They are defined by

(13) $$L\left(\phi \right)≜div\left(\frac{\nabla \phi }{\left|\nabla \phi \right|}\right)\delta_{\epsilon }\left(\phi \right),$$

(14) $$A\left(\phi \right)≜g\delta_{\epsilon }\left(\phi \right),$$

and

(15) $$V\left(\phi \right)=\kappa \delta_{\epsilon }\left(\phi \right)$$

where *g* is a positive and decreasing edge-detector function [11], which is defined by

(16) $$g\left(I\right)=\frac{1}{1+|\nabla G_{\sigma }{\;*\;I|}^{2}}$$

The convolution $G_{\sigma }*I$ is the smoother of *I* to reduce the noise of the image. The Gaussian kernel is defined by $G_{\sigma }\left(x,y\right)=\sigma^{-\frac{1}{2}}e^{\frac{-|x^{2}+y^{2}|}{4\sigma }}$, with a standard deviation σ. Actually, A(ϕ) is a weighted area term [30]. When the contour is away from the object boundary, it drives the contour to move towards the boundary. Contrarily, it slows down the shrinking or expanding of the contour ϕ.

The Heaviside function $H_{\epsilon }\left(\phi \left(x\right)\right)$ and Dirac delta function $\delta_{\epsilon }\left(\phi \left(x\right)\right)$ are chosen in the form of smooth approximation, defined as follows:

(17) $$H_{\epsilon }\left(x\right)=\left\{\begin{array}{cc} \frac{1}{2}(1+\frac{x}{\epsilon }+\frac{1}{\pi }sin\left(\frac{\pi (x)}{\epsilon })\right), & |x|\leq \epsilon \\ 1, & x>\epsilon \\ 0, & x<-\epsilon \end{array}\right.$$

and

(18) $$\delta_{\epsilon }\left(x\right)=\left\{\begin{array}{cc} \frac{1}{2\epsilon }\left(1+cos\left(\frac{\pi x}{\epsilon }\right)\right), & |x|\leq \epsilon \\ 0, & |x|>\epsilon \end{array}\right.$$

When the contour ϕ is far away from the object to be segmented and in the convex areas (s(κ)=1), $F(\phi ,c_{1},c_{2})$ plays the main role to drive the contour line towards the object boundary. With the curvature term V(ϕ) and s(κ) function, we can constrain the contour line ϕ to maintain convexity when it tends to be concave (κ<0) near the overlapping region.

The corresponding evaluation model can be expressed as follows:

(19) $$\begin{array}{cc} \frac{\partial \phi }{\partial t}=s\left(\kappa \right)\cdot \left[\lambda_{1}{\left(I-c_{1}\right)}^{2}-\lambda_{2}{\left(I-c_{2}\right)}^{2}\right]\delta_{\epsilon }\left(\phi \right) & +\alpha \cdot div\left(\frac{\nabla \phi }{\left|\nabla \phi \right|}\right)\delta_{\epsilon }\left(\phi \right)+\mu \cdot g\delta_{\epsilon }\left(\phi \right)\\ \; & +\left(1-s\left(\kappa \right)\right)\cdot \kappa \delta_{\epsilon }\left(\phi \right) \end{array}$$

We can see that, at the positions where κ≥0 (convex), the data-driven term $F(\phi ,c_{1},c_{2})$ plays the dominant role, while at the positions where κ<0 (concave), the curvature term V(ϕ) leads the motivation of the contour ϕ to the single organoid’s boundary. The length term L(ϕ) and area term A(ϕ) have been working throughout the evolution of ϕ, which control the smoothness and propagation of the contour ϕ.

To implement the contours evolving toward the object boundary, we can use the following iteration:

(20) $$\frac{\phi^{k+1}-\phi^{k}}{\Delta t}=R\left(\phi^{k}\right)$$

Here, *k* and Δt are the iteration number and time step and $R(\phi^{k})$ is the numerical approximation of the derivative terms in (19). Δt is limited by the Courant–Friedrichs–Lewy (CFL) Condition [31] to avoid oscillations occurring.

## 4. Automatic Initialization

Active contour models always need to determine an appropriate initial contour, and its selection of has a significant influence on the segmentation results. During the actual segmentation of the tumor organoid images, the initial contour’s position is manually adjusted for each new image. Especially for segmenting the two partially overlapping organoids in the same image, the initial contour should be adjusted separately. Therefore, we propose a human-computer interaction method to automatically generate initial contours for organoid images. Only one point at the center of the organoid that we want to divide that needs to be manually clicked, and the initial contour will be automatically generated. This method suits a single organoid image and a multiple overlapping organoid image. The advantage of this method is that there is little manual intervention, which significantly reduces labor costs, and we can freely choose the individual organoid we want to segment to generate the initial contour.

**Figure 2:**
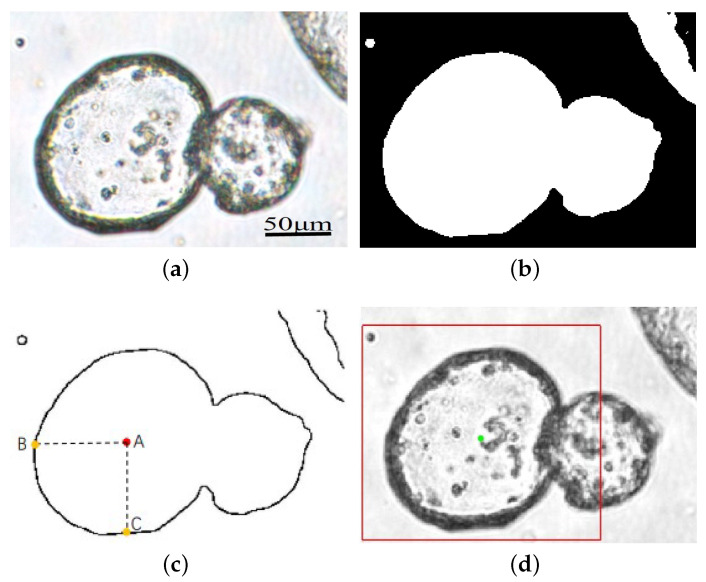
Generation process of the initial contour. (**a**) Original image. (**b**) Preliminary segmentation of the CV model. (**c**) Edge-detection results based on Canny operator. *A* is the inner center point, and *B* and *C* are the closest points to *A* horizontally and vertically, respectively. (**d**) The initial contour. The green point is the inner center point, and the red rectangular line is the initial contour.

**Figure 3:**
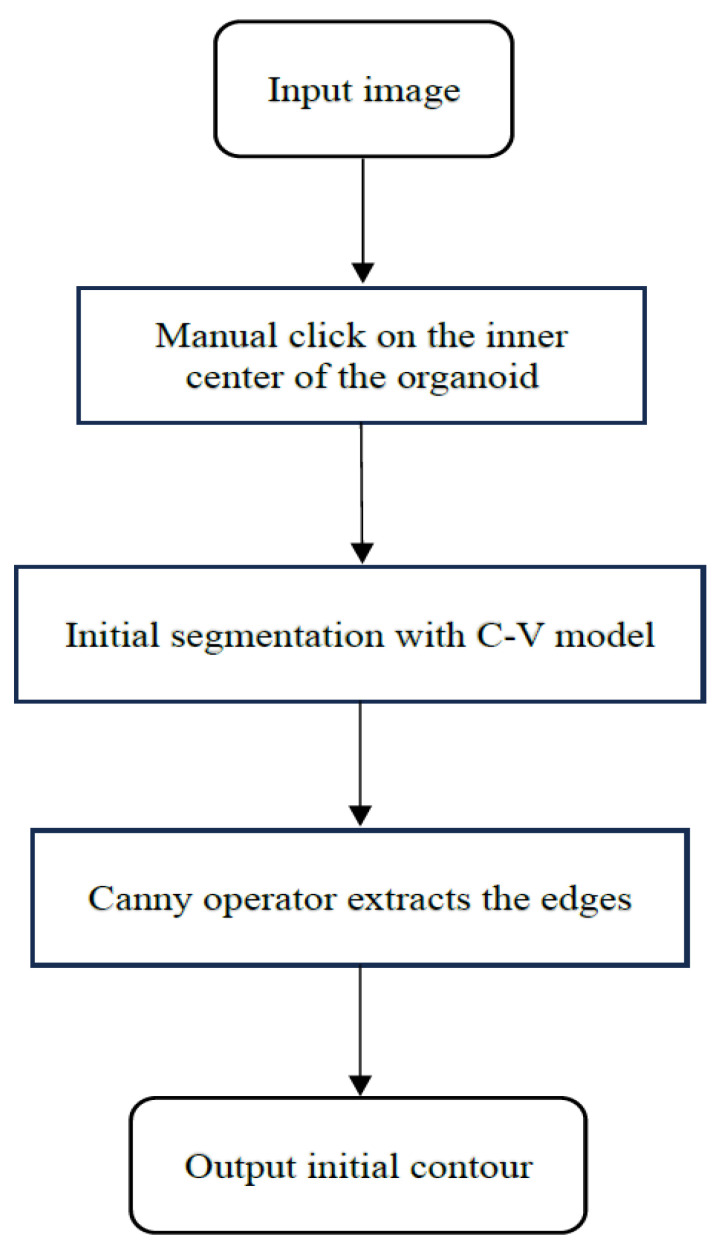
Algorithm flowchart of the automatic initialization.

For multiple tumor organoids in one image, click the inner center point of the organoid to be segmented, the initial contour of this organoid will be automatically generated. Let us take Figure 2a as an example to detail generating the initial contour. As shown in Figure 2a, it is an image of two overlapping tumor organoids. In the upper right corner is another tumor organoid. At first, use the C-V model to segment the entire image and obtain Figure 2b, then apply the Canny operator to extract its edges. We can see that the outer contour of the two overlapping organoids is visible, as shown in Figure 2c. Mark the inner center point as *A* and find the pixels *B* and *C* closest to point *A*’s horizontal and vertical directions in the outline pixel set of (c). Then, expand the length of AB and AC by 20 percent; it can form a rectangular contour line centered on *A*. As shown in Figure 2d, the green point is the center point we chose, and the red rectangle is the generated initial contour. Figure 3 is an algorithm flowchart that shows the critical steps of the initial contour-generation process more clearly.

With the above process, we realized that, during the actual segmentation process, there is no need to adjust the initial contour’s position manually. Only one point should be pointed at the inner center of the organoid to be segmented, and its initial contour can be automatically generated. On the other hand, the automatic initialization method proposed can also be applied to the segmentation of other circular objects, especially images with homogeneous background intensity.

## 5. Experiments and Results

In this section, the experimental results of the proposed model and comparisons with the existing models will be presented in detail. All methods were processed using a laptop with a 64-bit Windows 11 Enterprise, core i5 processor, and 16 GB of RAM, using the *Matlab2019b*. In this paper, the data set contains pancreatic ductal adenocarcinoma organoid images, which are provided by the School of Life Sciences, Nanjing University, China.

There are parameters, α, μ, β, $\lambda_{1}$, and $\lambda_{2}$, in our proposed model. α, μ, and β are the parameters of the length term, area term, and curvature term, respectively. $\lambda_{1}$ and $\lambda_{2}$ are the parameters of the data-driven term, which are the weights of the mean intensity inside and outside the contour, respectively. The model is not sensitive to the choice of the length term parameter α, which can be fixed for most applications. Nonzero $\lambda_{1}$ and μ give the internal and additional external forces to drive the motion of the contour. When the value of μ is positive, the contour evolves inward, and when the value is negative, the contour evolves outward. Consider that $\lambda_{1}$ and μ have the same effect and that we fixed the value of μ. β determines the effect of the curvature term; as its value increases, the effect of the curvature term becomes stronger when the contour becomes concave. Unless otherwise specified, these parameters are fixed as α=1, μ=10, and $\lambda_{2}=1$ in this paper. In the proposed model, only two parameters $\lambda_{1}$ and β need to be adjusted for different images. With a large number of experiments, we have found that, only for a few images with very unclear boundaries of the tumor organoid to be segmented, it is necessary to increase the value of β to prevent boundary leakage. However, for most images with clear boundaries, we can directly set β=1 and only tune the value of $\lambda_{1}$. As an important parameter in the data-driven term, the larger the value of $\lambda_{1}$, the stronger the ability to pull the contour inward and contract to the boundary of the organoid is. But, for images with blurred boundaries, the value of $\lambda_{1}$ should not be too large to prevent boundary leakage. In Appendix A, we also explain the effect of the values of each variable on the segmentation results.

According to the characteristics of the tumor organoid images, the internal intensity of the tumor organoid is very inhomogeneous, but the background is relatively intensity-homogeneous. Therefore, we selected the initial contour outside the tumor organoid and evolved it inward to shrink to the object boundary. When $\lambda_{1}=\lambda_{2}=1$, the data-driven term is equivalent to the C-V model. Consequently, the contour cannot overcome the influence of the overlapping tumor organoids, as shown in Figure 1b. Therefore, in order to segment the tumor organoid above, we should increase the value of $\lambda_{1}$ to pull the contour to shrink toward the boundary. On the other hand, a too-large a value of $\lambda_{1}$ may cause boundary leakage for images with weak boundaries. Therefore, we need to choose the appropriate $\lambda_{1}$ to achieve a satisfactory segmentation result. Empirically, for common organoid images, the optimal value of $\lambda_{1}$ is between 1 and 5.

**Figure 4:**
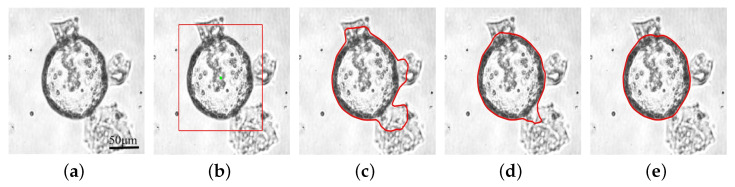
An example of the iterative process of our model. (**a**) The original image. (**b**) The inner center point and the generated initial contour. (**c**,**d**) Intermediate iterations of 200 and 800. (**e**) Final result. The red lines show the position of the contours.

Figure 4 shows an example of the curve evolution process using our method. For this image, we made $\lambda_{1}=3.5$. Figure 4b presents the initial contour automatically generated by manually selecting the center point (green point) of the organoid using the method mentioned above. Figure 4c,d show the shapes of the contour at 200 and 800 iterations, and Figure 4e is the final result. The difficulty in segmenting this image is making the contour overcome the impact of three parts of the impurities in the culture dish and shrink towards the organoid boundary with the convexity-preserving ability. We can see that our model has a strong convexity-preserving force so that it can pull the contour through those impurities and contract towards the organoid boundary, as shown in Figure 4c,d. On the other hand, the convexity-preserving ability also ensures that, when the contour shrinks to the vicinity of the boundary, it does not cross the boundary and enter the organoid’s interior, effectively avoiding boundary leakage.

Our model has been compared with the C-V and CPLSE models regarding how precisely and rapidly they segment organoids to evaluate the segmentation performance. The quantitative evaluation of these models is obtained by calculating the Dice value on their segmentation results, and the Dice score is defined as follows:

(21) $$Dice=\frac{2|A⋂G|}{\left|A|+|G\right|}$$

where |·| stands for the region area, *A* is the segmentation results, and *G* is the ground truth.

We selected 51 pancreatic ductal adenocarcinoma organoid images with typical segmentation difficulties to illustrate the segmentation results. In our experiment, the ground truth was obtained from experienced physicians. The average Dice values of C-V, CPLSE, and our model were 84.84±8%, 92.21±4.42%, and 98.81±0.48%, as shown in Table 1. We can see that the proposed model has significantly improved the segmentation accuracy of tumor organoids. It has a very small standard deviation, indicating that the segmentation results are very stable. As for the time efficiency, the average computation time of our model is 20.67 s, which is comparable to the C-V model (20.43 s), while the CPLSE model takes 28.63 s. Our model is 1.4-times faster than the CPLSE model. The comparative details are shown in Table 1, where i represents the average of the total iteration numbers and T represents the average of the total computational time.

**Figure 5:**
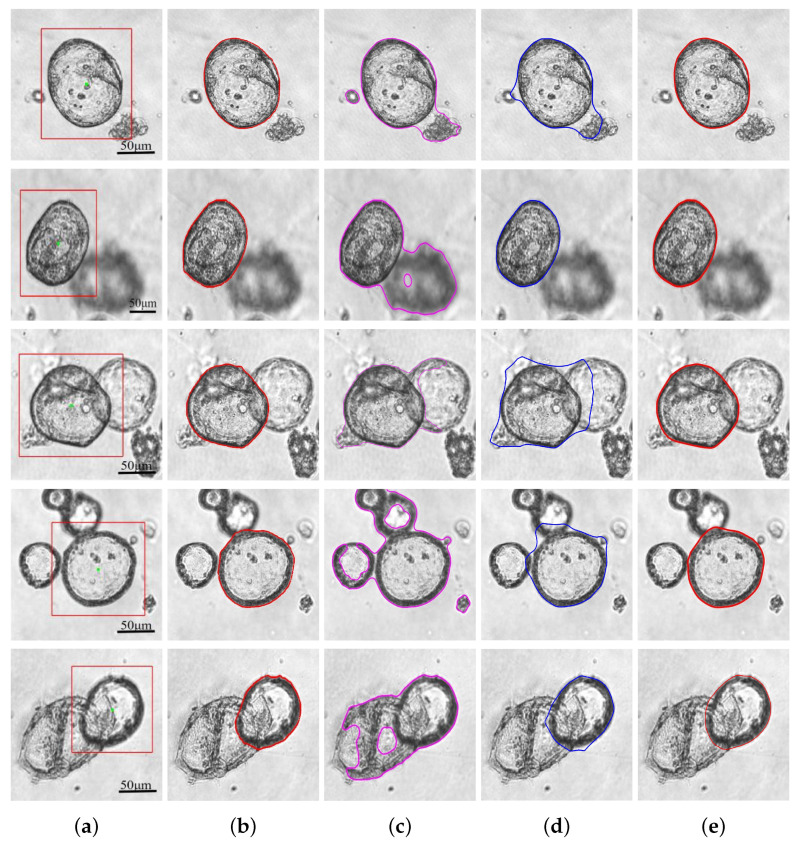
Comparison results of five organoid images. (**a**) Original images and initial contours. The green point is the inner center point, and the red rectangular line is the initial contour. (**b**) Ground truth. (**c**–**e**) Results of C-V model, CPLSE model, and proposed model. The lines of different colors show the position of the contours of the different models.

We selected five typical images with different characteristics to show the segmentation results of different methods, as shown in Figure 5. Each row corresponds to one image. The first column is the input images with the generated initial contours, and the second column is the ground truth labeled by experienced physicians. The third, fourth, and fifth columns are the segmentation results of C-V, CPLSE, and our model, respectively. It is obvious that the C-V model will segment almost all boundaries in the image, which is not satisfactory. Although the CPLSE model can preserve convexity and make the evolution of the contour tend to shrink to the organoid boundaries, it is still easily influenced by other distractions, as shown in Figure 5; only for the second image has CPLSE achieved satisfactory results, and the remaining images cannot be accurately segmented. In contrast, our model can achieve satisfactory results for every image. If we choose appropriate parameters, we can accurately segment organoids. Table 2 presents each image’s Dice values and computation time. we can see that our model has a much higher Dice value than the other two models; on the other hand, the computation time is also significantly improved. For images 1, 3, and 5 in Figure 5, we made $\lambda_{1}=3.5$. For image 2, since the intensity of the shadow is similar to the background, $\lambda_{1}$ can be taken as a small value, $\lambda_{1}=2.5$. For image 4, the influencing factor is the other organoid boundaries on the left and upper sides, and then, we chose $\lambda_{1}=3$. For these five images, we set β=1 for all.

## 6. Discussion and Conclusions

This paper proposes an innovative level-set evolution model for tumor organoid image segmentation combined with a convexity-preserving ability. Organoids are three-dimensional cell clusters cultivated in culture dishes. A common problem in organoid image segmentation is the separate segmentation of the overlapping organoids. The proposed model solves this problem and can accurately achieve convex segmentation of individual tumor organoids. The total evolution model for ϕ consists of a data-driven term, a length term, an area term, and a curvature term. The data-driven and curvature terms are incorporated with the convex indicator, respectively. When ϕ is convex, the data-driven term works, and the curvature term fails. When the contour moves near the boundary of the organoid and is influenced by other substances, ϕ tends to be concave, the data-driven term becomes invalid, and the curvature term takes effect, pulling the contour towards convexity. The area and length terms work throughout the entire segmentation process, maintaining the excellent properties of the contour and providing balloon force. By utilizing the proposed model, we were able to obtain segmentation results with an average Dice value of 98.81±0.48%.

For active contour methods, the position of the initial contour plays a significant role in determining the accuracy of the segmentation results. Organoids are usually cultivated in culture dishes, resulting in a relatively homogeneous background of organoid images that are less affected by noise or artifacts, but the intensity inside the organoids is highly inhomogeneous. Therefore, finding the initial contour outside the organoid and then shrinking it inward are necessary. To simplify this process, we have developed an automatic initialization method that requires only one click on the inner center of the organoid to be segmented. We used the C-V model to initially segment the tumor organoid image and the Canny operator to detect edges and determine the initial contour of the organoid. This approach reduces the labor costs and speeds up the segmentation process significantly. During the segmentation process, the interference of overlapping areas on the contour evolution process is much greater than the noise or artifacts. The proposed convexity-preserving segmentation model can pull the contour to overcome the influence of overlapping regions and contract inward to the boundary of the organoids.

According to the experimental results, our model can achieve high-quality segmentation results with fast computational speed. Compared to the C-V and CPLSE models, our model shows significant improvement in the Dice value, all above 95%, which is very close to the ground truth. In Figure 5, it can be observed that the C-V model tends to segment the overall boundary, while the CPLSE model struggles to overcome the influence of other organoids, and only the second image has a satisfactory segmentation result. In the case of the second image, the blurred shadow on the right side of the organoid to be segmented is another organoid not in the focal plane, and the difference in intensity between this shadow and the background is not particularly large, leading to a good segmentation result for the CPLSE model. However, for images 1, 3, and 4, the CPLSE model cannot overcome external influences, and the convexity-preserving ability is not strong enough to shrink the contour to the boundary of the organoid. For image 5, the contour has not shrunk on the lower and left sides of the organoid to be segmented, but the right side has already crossed the edge. Conversely, our proposed model overcomes the influence of other organoids and impurities and has excellent convexity-preserving ability. It achieves an average Dice value of 98.67% and shows excellent segmentation results on every image.

In conclusion, based on tumor organoid image characteristics and segmentation difficulties, we propose an algorithm with robust convexity preservation for the segmentation of tumor organoid images. It does not require a large data set compared to deep learning methods. In addition, an automatically generated initialization is designed so that the convexity-preserving segmentation algorithm is simple and easy to perform for the selected tumor organoid. Numerical experiments show that the algorithm can segment individual target tumor organoids quickly and efficiently on complex organoid images, which is crucial for subsequent property analysis in tumor organoid studies. Some aspects of the model can be improved. On the one hand, due to the characteristics of organoid images, the initial contour needs to be located outside the target organoid, resulting in a unidirectional contour evolution. On the other hand, the data of organoid images are limited, and we only conducted model validation on small sample of data. In the future, we will continue to explore the improvement of the proposed segmentation model and methods for determining the optimal values of parameters. If the organoid image data are accessible, we will apply our model to a big data set. 

## Figures and Tables

**Table 1:** Average computation time and Dice values of 51 images of the three methods.

	i	T	Dice Value
CV	1234	20.43 s	84.84±8%
CPLSE	1213	28.63 s	92.21±4.42%
Proposed	1162	20.67 s	98.81±0.48%

**Table 2:** Dice values and computation time for the five images of the three methods.

	1	2	3	4	5	Average of 5 Images
C-V	91.09_(38 s)_	68.84_(19 s)_	91.43_(23 s)_	77.52_(47 s)_	69.00_(43 s)_	79.58_(34 s)_
CPLSE	93.06_(77 s)_	98.26_(65 s)_	83.67_(78 s)_	92.87_(88 s)_	95.83_(190 s)_	92.74_(100 s)_
Proposed	99.27_(44 s)_	98.88_(9 s)_	99.17_(31 s)_	98.63_(12 s)_	97.39_(14 s)_	98.67_(22 s)_

## Data Availability

The data presented in this study are available upon request from the corresponding author.

## Abbreviations

The following abbreviations are used in this manuscript:

ACM	active contour model
C-V	Chan–Vese
CSI	curvature sign indicator
DRLSE	distance-regularized level-set evolution
CPLSE	convexity-preserving level-set evolution

## Appendix A

The proposed model contains five parameters, $\lambda_{1}$, $\lambda_{2}$, α, μ, and β. The selection of the parameter value is related to the segmentation performance. In the segmentation experiment of pancreatic ductal adenocarcinoma organoid images in this article, only the parameters $\lambda_{1}$ and β need to be adjusted. For most images, β can be set to 1, and only the value of $\lambda_{1}$ needs to be adjusted. Table A1 provides a detailed introduction to the values of each parameter and their impact on the segmentation performance of the model.

**Table A1:** Detailed description of parameters in the model.

Parameter	Value	The Impact on Segmentation Results
$\lambda_{1}$	1–5	It is a parameter of the average grayscale inside the contour. The larger is value, the stronger the ability of the contour to evolve inward to the boundary of the organoid. For images with blurred boundaries, the value of $\lambda_{1}$ should not be too large.
$\lambda_{2}$	1	It is the parameter of the average grayscale outside the contour. In order to highlight the role of $\lambda_{1}$, $\lambda_{2}$ is set to a fixed value of 1, and the value of $\lambda_{1}$ is set to be greater than 1 to facilitate pulling the contour to shrink.
α	1	It is a parameter of the length term, and the model is not sensitive to the value of α, so it can be set to a fixed value of 1.
μ	10	It is a parameter of the area term, providing balloon forces for contour evolution. When d is positive, the contour shrinks inward. When d is negative, the contour expands outward. Due to the similar effect of μ and $\lambda_{1}$, we fixed the value of μ to 10.
β	1	It is a parameter of the curvature term. The larger its value, the stronger the constraint on the convexity of the contour. For most images, the value of β can be fixed to 1, and for some images with blurred boundaries or strong noise, the value of g can be increased.
